# Antioxidation and Melanogenesis Inhibition of Various *Dendrobium tosaense* Extracts

**DOI:** 10.3390/molecules23071810

**Published:** 2018-07-21

**Authors:** Chin-Feng Chan, Chin-Tung Wu, Wen-Ying Huang, Wen-Shin Lin, Han-Wei Wu, Teng-Kuan Huang, Min-Yun Chang, Yung-Sheng Lin

**Affiliations:** 1 Department of Applied Cosmetology, Hung-Kuang University, Taichung 43302, Taiwan; cfchanjames@hotmail.com (C.-F.C.); beca690420@hk.edu.tw (W.-Y.H.); 2 Bachelor Program in Interdisciplinary Studies, College of Future, National Yunlin University of Science and Technology, Yunlin 64002, Taiwan; ctwu5@yuntech.edu.tw; 3 Department of Plant Industry, National Pingtung University of Science and Technology, Pingtung 91201, Taiwan; wslin@mail.npust.edu.tw; 4 Ying Li Biotech Co., Ltd., Taichung 40342, Taiwan; pgrace10171@gmail.com; 5 Department of Chemical Engineering, National United University, Miaoli 36003, Taiwan; qwe1101237@gmail.com (T.-K.H.); mino@nuu.edu.tw (M.-Y.C.)

**Keywords:** *Dendrobium tosaense*, polyphenol, antioxidation, mushroom tyrosinase, B16F10, melanogenesis

## Abstract

This study investigated the polyphenol content, antioxidant activity, and inhibition ability of mushroom tyrosinase and melanogenesis of *Dendrobium tosaense* (DT) extract. Ground DT was extracted using deionized water (W) or 50% ethanol (50E) at room temperature (RT) or 50 °C (50T) for 20 min. The 50T + 50E extract exhibited the highest total phenol content 47.0 ± 4.0 mg gallic acid equivalent/g DT extract, the highest level of 2,2′-azino-bis(3-ethylbenzothiazoline-6-sulphonic acid) free-radical scavenging 66.0 ± 3.0 mg Trolox equivalent/g DT extract, and the highest reducing power 12.00 ± 0.50 mg vitamin C equivalent/g DT extract. The RT + W extract had the highest total flavonoid content 110.0 ± 3.0 mg quercetin equivalent/g DT extract. The RT + 50E extract had the lowest half maximal inhibitory concentration 1.30 ± 0.00 mg/mL for 2,2-diphenyl-1-picrylhydrazyl free-radical scavenging, and the lowest half maximal inhibitory concentration 6.40 ± 0.30 mg/mL for mushroom tyrosinase inhibition activity. DT extracts, especially RT + W and 50T + W, exhibited potent inhibitory effects on melanogenesis of B16/F10 cells. These results demonstrated the application potential of DT extract for skincare.

## 1. Introduction

The origins of orchids (Orchidaceae) can be traced back 120 million years [1]. *Dendrobium*, known as “Shihu” in China, is the second largest genus in Orchidaceae [1]. Most *Dendrobium* species are epiphytic and grown in tropical and subtropical regions [2]. *Dendrobium* serves numerous functions in traditional Chinese medicine, including as an antipyretic, as an anti-inflammatory agent, for benefit of the eyes and digestive system, and for lowering blood sugar [3]. *Dendrobium* has also attracted attention for its potential inhibition and prevention of cancer, its immune-system stimulation, and its antioxidant activity [4]. *Dendrobium tosaense* (DT), *Dendrobium linawamum* (DL), and *Dendrobium moniliforme* (DM) are well-known medicinal plants, but DT and DM are more commonly used than DL [5]. DT is native to Taiwan and can be mass-produced through tissue culture [6]. 

The chemical compounds in the *Dendrobium* species include alkaloids, bibenzyls, fluorenones, phenanthrenes, sesquiterpenoids, trace elements, polysaccharides, and amino acids [1,5,6,7,8,9]. Dendrobine is most prevalent among the alkaloids and may combat cardiovascular disease and gastrointestinal disorders, in addition to exhibiting antitumor, analgesic, and antipyretic properties [10,11]. Gigantol is a type of bibenzyl compound [12] that functions as an antioxidant and contributes to inhibition of cataractogenesis as well as exhibiting anti-lung-cancer and anti-platelet-aggregation properties [1,13]. *Dendrobium* is also rich in active polysaccharides, which exhibit antitumor and antioxidation properties and contribute to enhanced immunity [14]. 

Tyrosinase acts as the critical enzyme in the melanin biosynthesis pathway [15,16,17]. Therefore, anti-melanogenic activity can be prescreened via tyrosinase inhibition. However, many melanogenesis inhibitors do not exhibit inhibitory effects on mushroom tyrosinase activity [16,18,19]. Accordingly, cell-based assays should also be further studied to investigate melanogenesis inhibition in vivo. Many natural plant products catch scientists’ attention for becoming new skin-whitening materials [18,20,21,22]. However, there are few reports about the evaluation of DT for melanogenesis inhibition.

Solvent extraction is a simple and efficient technique for obtaining extracts from plants. However, few reports have evaluated DT extracts that have been prepared through various methods. Therefore, the aim of the present study was to determine the polyphenol contents, antioxidant activity, mushroom tyrosinase inhibition ability and melanogenesis inhibition of DT samples obtained using various solvent extraction methods.

## 2. Results and Discussion

### 2.1. Liquid Chromatography Coupled with Tandem Mass Spectrometry (LC/MS/MS) Analysis

**Figure 1:**
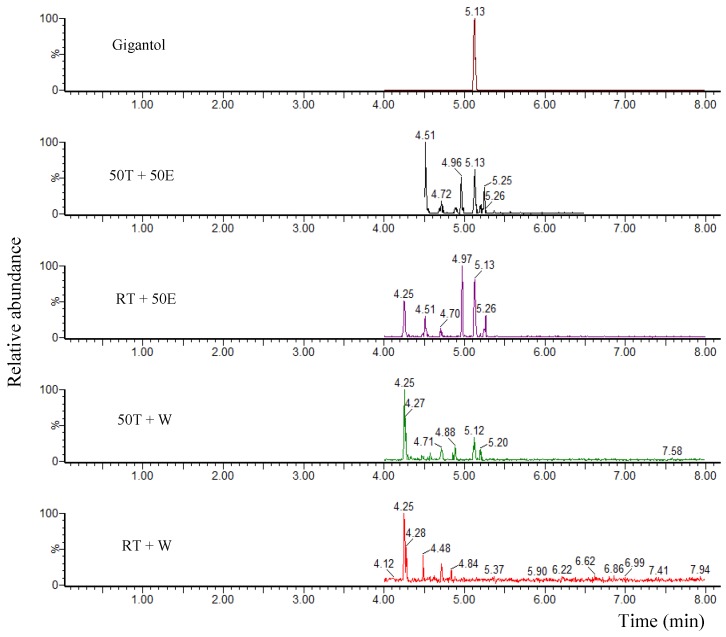
LC/MS/MS chromatograms of gigantol in the four extracts.

Gigantol in four extracts was identified through mass spectrometry as illustrated in Figure 1. A peak in retention time at 5.13 min with a [M − H]^−^ molecular ion at *m*/*z* 273 was detected for all extracts except the room temperature plus water (RT + W) extract. The order of samples according to amounts of gigantol from high to low level was RT + 50E, 50T + 50E, and 50T + W.

### 2.2. Total Phenol Content

**Figure 2:**
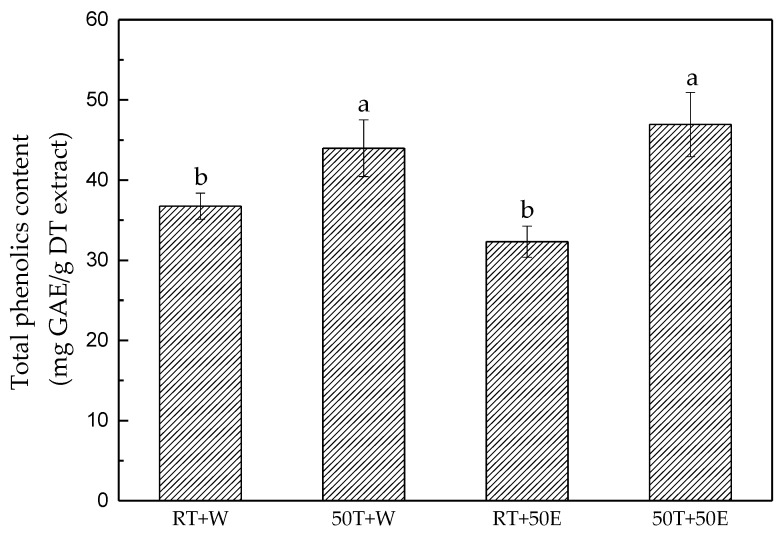
Total phenol content for the four extracts. Treatment means with the same letter are not significantly different.

Phenolics have significant antioxidation and anticancer effects and contribute to prevention of cardiovascular diseases. The experimental results indicate that the 50T + 50E extraction had the highest total phenol content, at 47.0 ± 4.0 mg gallic acid equivalent/g DT extract (Figure 2). Phenolic compounds are more effectively extracted at an elevated temperature, which enhances both the diffusion coefficient and the solubility of polyphenols [23,24]. Additionally, the aqueous extraction may lead to an extract with a high percentage of impurities that interfere in determination of phenolic compounds [25,26]. 

### 2.3. Total Flavonoid Content

**Figure 3:**
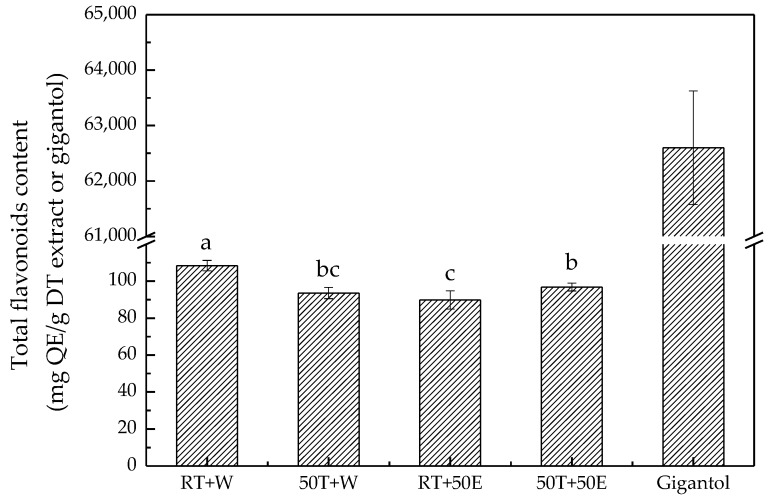
Total flavonoid content for the four extracts and gigantol. Treatment means with the same letter are not significantly different.

Flavonoids have more than 3000 varieties and are potent antioxidants with free-radical-scavenging capabilities [27]. As depicted in Figure 3, RT + W extract in this study exhibited the highest total flavonoid content, at 110.0 ± 3.0 mg quercetin equivalent/g DT extract. Flavonoids are water-soluble ingredients, and degradation of flavonoids occurs at relatively high temperatures [28]. The lower total flavonoid content at higher temperatures may also be related to the structures of particular flavonoids [28]. These properties explain the study results, which indicated that a lower temperature resulted in an extract with higher total flavonoid content.

### 2.4. DPPH Free-Radical-Scavenging Assay

**Figure 4:**
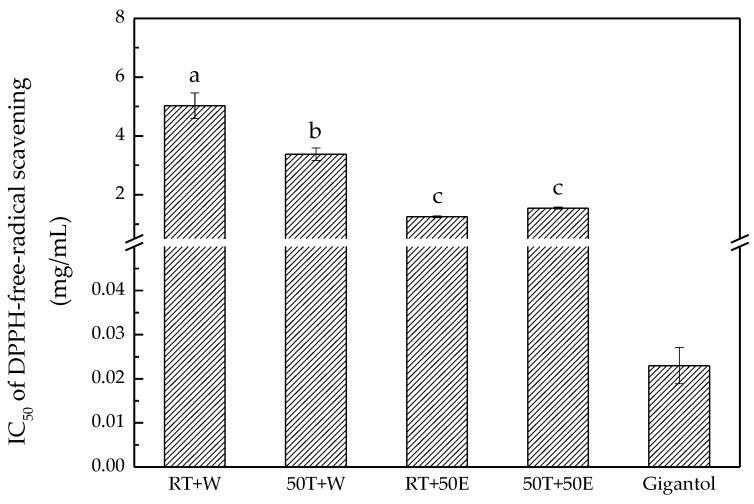
DPPH free-radical scavenging for the four extracts and gigantol. Treatment means with the same letter are not significantly different.

This study demonstrated that DT extract decreased the absorbance of DPPH solution. The decrease in absorbance of DPPH solution most likely resulted from the reaction of antioxidants and DPPH through hydrogen donation [29]. Polysaccharides play a critical role in free-radical scavenging and contribute to DT’s favorable DPPH free-radical-scavenging ability [13]. In this research, the experimental results indicated that the RT + 50E extract had the lowest half maximal inhibitory concentration (IC_50_), 1.30 ± 0.00 mg/mL for DPPH (Figure 4). The IC_50_ of the RT + W, 50T + W, and 50T + 50E extracts were 5.00 ± 0.40, 3.40 ± 0.20, and 1.50 ± 0.00 mg/mL, respectively.

### 2.5. 2,2′-Azino-bis(3-Ethylbenzothiazoline-6-Sulphonic acid) (ABTS) Free-Radical Scavenging Assay

**Figure 5:**
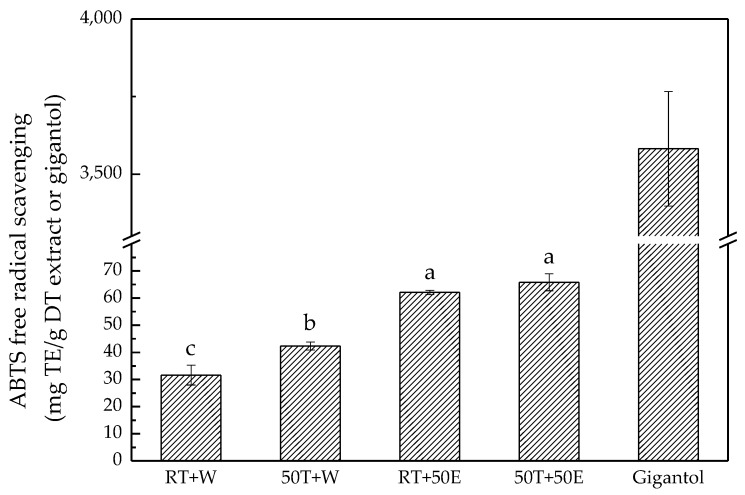
2,2′-Azino-bis(3-Ethylbenzothiazoline-6-Sulphonic acid) (ABTS) free-radical scavenging for the four extracts and gigantol. Treatment means with the same letter are not significantly different.

ABTS assay is often used in evaluating the total antioxidant power of plants [4]. In this study, the 50T + 50E extract exhibited the highest level of ABTS free-radical scavenging, at 66.0 ± 3.0 mg Trolox equivalent/g DT extract (Figure 5). This ABTS free-radical-scavenging assay revealed similar results to those obtained from the DPPH free-radical-scavenging assay [30]. The finding that DT extracted with 50% ethanol exhibited better DPPH and ABTS free-radical-scavenging abilities than DT extracted with water was consistent with previous findings for *Dendrobium* flower extracts [31].

### 2.6. Ferric-Reducing Power Assay

**Figure 6:**
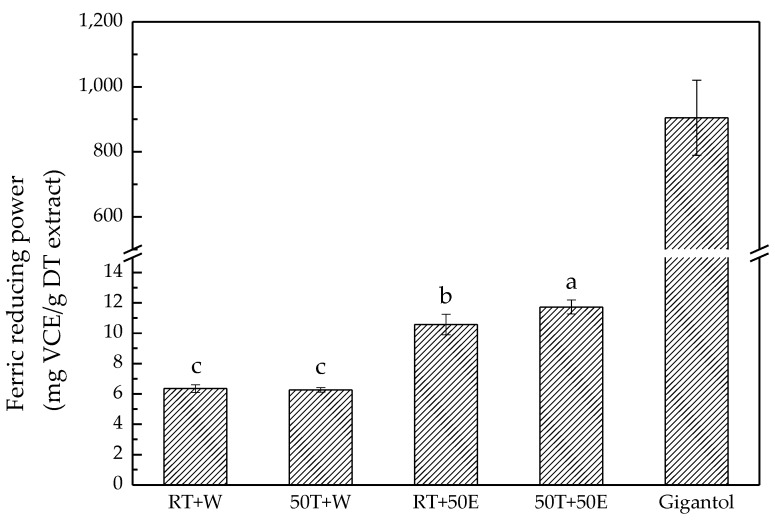
Ferric-reducing power of the four extracts and gigantol. Treatment means with the same letter are not significantly different.

In this assay, all extracts exhibited the same ability to reduce Fe^3+^/ferricyanide complex to Fe^2+^ through electron donation. The experimental results revealed that the 50T + 50E extract exhibited the highest ferric-reducing power, at 12.00 ± 0.50 mg vitamin C equivalent/g DT extract (Figure 6). The extracts obtained using 50% ethanol exhibited higher ferric-reducing power than those obtained with water. The results obtained for extraction with 50% ethanol at approximately 50 °C corresponded to findings in a previous study regarding the optimal condition in response surface methodology for the extraction of rice bran [32].

### 2.7. Mushroom Tyrosinase Inhibition Activity Assay

**Figure 7:**
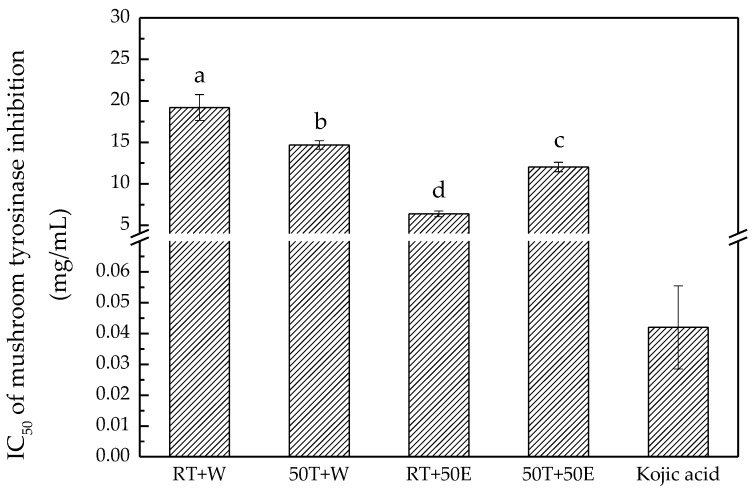
Mushroom tyrosinase inhibition ability of the four extracts and kojic acid. Treatment means with the same letter are not significantly different.

In the melanogenesis process, tyrosinase converts l-tyrosine into l-dihydroxyphenylalanine (l-DOPA) and then oxidizes l-DOPA to form dopachrome, which can be converted into melanin. Therefore, inhibition of tyrosinase is generally evaluated in the process of screening potential inhibitors of melanogenesis [33]. In this research, the experimental results indicated that the RT + 50E extract exhibited the strongest mushroom tyrosinase inhibition ability, at IC_50_ 6.40 ± 0.30 mg/mL (Figure 7). The extracts obtained using 50% ethanol exhibited higher mushroom tyrosinase inhibition activity than those obtained with water. These results were similar to those regarding DPPH and ABTS free-radical scavenging. 

### 2.8. Cell Viability and Melanin Content Assessment

**Figure 8:**
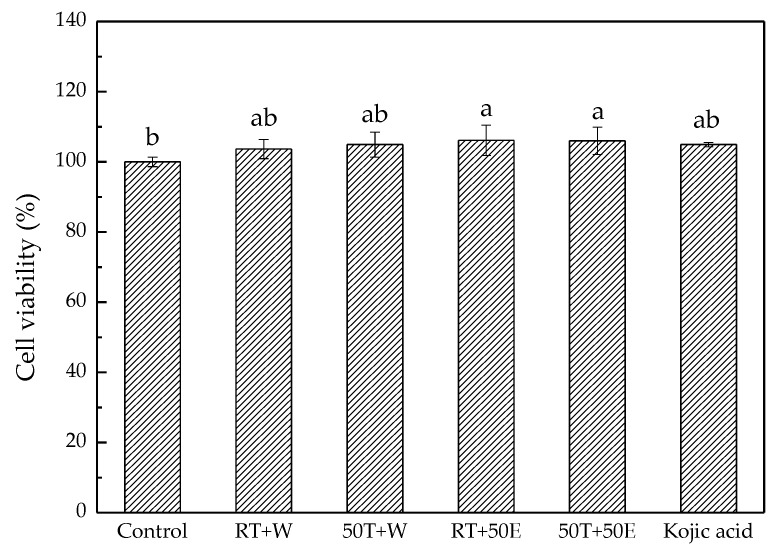
Cell viability of B16/F10 cells treated with the four extracts and kojic acid. Treatment means with the same letter are not significantly different.

**Figure 9:**
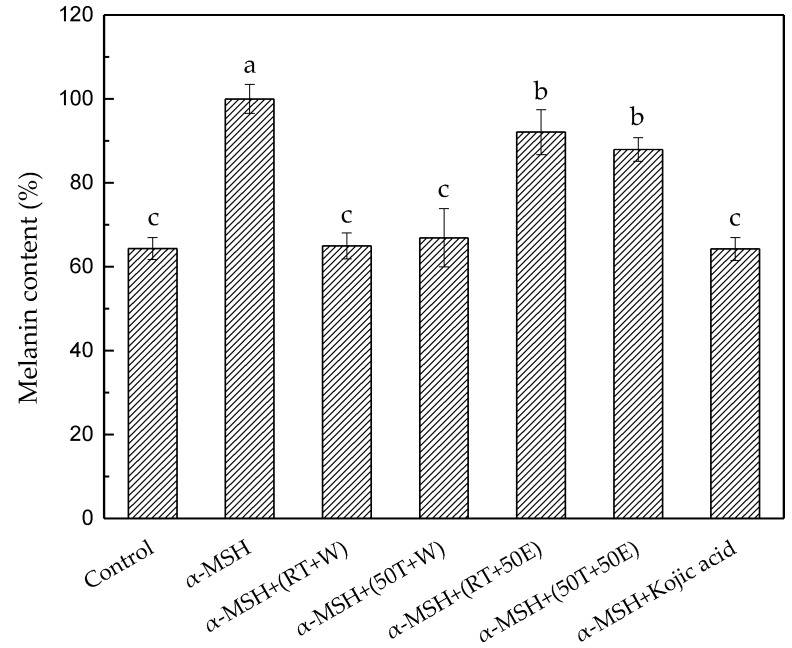
Melanin content in B16/F10 cells treated with the four extracts and kojic acid. Treatment means with the same letter are not significantly different.

In the methylthiazol tetrazolium (MTT) assays (Figure 8), all DT extracts and 1 mg/mL of kojic acid exhibited no cytotoxicity to the B16/F10 cells. Figure 9 shows the α-MSH-mediated melanogenesis inhibitory effects of DT extracts which were determined by calculating the amount of intracellular melanin in the presence of α-MSH. Compared with the group treated with α-MSH alone, RT + W and 50T + W significantly reduced the α-MSH-induced cellular melanin content, and were almost as effective as the positive control, kojic acid. The inhibitory effects of RT + W, 50T + W, and kojic acid on melanin production were 35.0 ± 3.0%, 33.0 ± 7.0%, and 36.0 ± 3.0%, respectively. The results of this study suggested that RT + W and 50T + W can substantially inhibit the melanogenesis on B16/F10 cells, and induce no cytotoxicity.

## 3. Materials and Methods 

### 3.1. Preparation of DT Extract

Stems of DT were powdered using a pulverizer. A total of 5 g of DT powder was extracted using 50 mL of deionized water (this method is labeled W) or 50% ethanol (50E) at room temperature (RT) or 50 °C (50T) for 20 min. After centrifugation with 3000 rpms for 10 min at room temperature and filtration, a liquid extract was obtained. The lyophilization process was further applied to obtain the dry extract to determine the concentration of DT extract. Finally, the concentrations of DT extract in original working solutions of RT + W, 50T + W, RT + 50E, and 50T + 50E were 6.23, 6.60, 4.38, and 4.70 mg/mL, respectively.

### 3.2. Liquid Chromatography–Tandem Mass Spectrometry (LC/MS/MS) Analysis

A liquid chromatography–tandem mass spectrometry system (UPLC-Xevo-TQMS) with a reverse-phase column (ACQUITY UPLC BEH C18, 1.7 μm, 2.1 × 100 mm^2^) was used for analysis. The mobile phase consisted of a mixture of 0.1% formic acid solution and acetonitrile containing 0.1% formic acid. The proportion of 0.1% formic acid in the mobile phase was 95% at 0 min, 90% at 0–2 min, 82% at 2–3 min, 62% at 3–4 min, 0% at 4–6 min, and 95% at 6–10 min. The flow rate was 0.35 mL/min. The MS/MS settings were a negative electrospray ion source, 3 kV capillary voltage, 3 V cone voltage, 350 °C desolvation temperature, and 0.15 mL/min collision gas flow.

### 3.3. Determination of Total Phenol Content

A homogeneous mixture of 200 μL of 0.5 N Folin-Ciocalteu phenol reagent and 200 μL of DT extract was obtained, and then 200 μL of 10% sodium carbonate and 400 μL of deionized water were added. The reaction was conducted in the dark at RT for 1 h. Subsequently, centrifugation with 7000 rpms for 15 min at room temperature was applied. A total of 200 μL of supernatant was collected and its absorbance measured at 700 nm using a spectrophotometer. Total phenol content was determined by gallic acid standard curve and expressed as milligram gallic acid equivalent per gram of DT extract.

### 3.4. Determination of Total Flavonoid Content

Mixture of 200 μL of DT extract, 80 μL of CH_3_OH, and 100 μL of 5% methanol was performed. The mixture was subsequently left at RT for 5 min, followed by addition of 20 μL of 10% aluminum chloride. After 6 min, 200 μL of 1 N sodium hydroxide was added. The reaction was conducted in the dark at RT for 1 h. The absorbance of the resulting mixture was measured at 510 nm. The total flavonoid content was evaluated from a quercetin standard curve.

### 3.5. Determination of 2,2-Diphenyl-1-Picrylhydrazyl (DPPH) Free-Radical Scavenging Activity

A homogeneous mixture of 500 μL of 95% ethanol solution of DPPH and 50 μL of DT extract was obtained and then left in the dark for 30 min. The absorbance of the resulting mixture was measured at 517 nm. Vitamin C was used as a standard, and pure water solution was used as the control.

(1) $$\mathrm{DPPH}\;\mathrm{free}-\mathrm{radical}\;\mathrm{scavenging}\;\mathrm{activity}\;(\%)=[1-(\frac{A\;517\;of\;sample}{A\;517\;of\;blank})]\times 100\%$$

### 3.6. Determination of 2,2′-Azino-bis(3-Ethylbenzothiazoline-6-Sulphonic acid) (ABTS) Free-Radical Scavenging Activity

A mixture of 250 μL of 7 mM ABTS and 250 μL of 2.45 mM potassium persulfate was obtained and incubated at 4 °C for 16 hours in darkness. The background absorbance was diluted to 0.7 ± 0.05 using 95% ethanol. A homogenous mixture of 180 μL of the adjusted mixed solution and 20 μL of DT extract was obtained and then incubated at RT for 10 min in darkness. The absorbance of the resulting mixture was measured at 734 nm. ABTS free-radical scavenging activity was evaluated from a Trolox standard curve.

### 3.7. Determination of Ferric-Reducing Power Activity

A mixture of 100 μL of 0.2 M phosphate-buffered saline (pH 6.6), 100 μL of 1% potassium ferricyanide, and 200 μL of DT extract was obtained and subjected to a water bath at 50 °C. After 20 min of reaction, the mixture was rapidly cooled for 3 min. After the addition of 100 μL of 10% trichloroacetic acid and the application of centrifugation with 7000 rpms for 10 minutes at room temperature, 400 μL of supernatant was withdrawn and added to 400 μL of deionized water and 100 μL of 0.1% iron chloride. After 10 min in the dark, Prussian blue formed in the mixture. The absorbance of the resulting mixture was measured at 700 nm. The ferric-reducing power was evaluated from a vitamin C standard curve.

### 3.8. Determination of Mushroom Tyrosinase Inhibition Activity

A 96-well plate was filled with 40 μL of 3 mM L-Dopa dissolved in 67 mM phosphate buffer at pH 6.8, 20 μL of DT extract, and 40 μL of mushroom tyrosinase solution (200 U). In this experiment, DT extract obtained using 50E was freeze-dried and then redissolved into dimethyl sulfoxide (DMSO). The mixture was homogenized and incubated at 37 °C for 30 min. The absorbance of the resulting mixture was measured at 475 nm for monitoring dopachrome which appeared during the reaction catalyzed by mushroom tyrosinase. Kojic acid was used as a standard, and pure water solution was used as the control.

(2) $$\mathrm{Mushroom}\;\mathrm{tyrosinase}\;\mathrm{inhibition}\;\mathrm{activity}\;(\%)=[1-(\frac{A\;475\;of\;sample}{A\;475\;of\;blank})]\times 100\%$$

### 3.9. Cell Viability

B16/F10 cells were cultured in Dulbecco’s minimum essential medium (DMEM) supplemented with 100 units/mL of penicillin G, 10% fetal bovine serum, 0.25 μg/mL of amphotericin, and 100 μg/mL of streptomycin, and then incubated at 37 °C with 5% CO_2_. The viability of cells treated with 20 μL of DT extract with a quintuple dilution was determined using a methylthiazol tetrazolium (MTT) assay. In brief, 3 × 10^4^ B16/F10 cells were seeded on 96-well plates. After 24 h, DMEM was removed, and 200 μL of fresh DMEM and DT sample were added. The cells were then incubated for 24 h. After incubation, the medium was removed, and 100 μL of MTT in a PBS solution (0.5 mg/mL) was added to each well. The cells were then incubated at 37 °C for 40 min. Subsequently, the MTT solution was removed, 100 μL of DMSO was added to each well, and the plate was gently shaken to dissolve the formazan crystals. The absorbance of each well was measured at 570 nm.

### 3.10. Assessment of Melanin Content

The cellular melanin content was determined as previously described [17]. In brief, 1 × 10^5^ B16/F10 cells were seeded in 24-well plates and cultured at 37 °C with 5% CO_2_ for 48 h. Subsequently, cells were treated with 200 nM α-MSH for 24 h, and then incubated with 20 mg/mL DT extract or 1 mg/mL kojic acid for 24 h. The cells were washed twice with PBS, and the cell pellets were then dissolved in 200 μL of 1 N NaOH in 10% DMSO for 1 h at 80 °C. The relative melanin content was determined by measuring the absorbance at 405 nm. The melanin content was calculated using the following equation, (absorbance of sample/absorbance of α-MSH) × 100%.

### 3.11. Statistical Analyses

The experiments were laid out in Completely Randomized Design (CRD) with three replications. The evaluated data are presented as the mean ± standard error. The data collected were analyzed by analysis of variance (ANOVA) using SAS version 9.4 (SAS Institute, Inc., Cary, NC, USA). If significant, means were separated by least significant difference (LSD) at *p* ≤ 0.05. Treatment means with the same letter are not significantly different at the 5% level.

## 4. Conclusions

In summary, this study confirmed that DT is rich in polyphenols and displays antioxidant and inhibition on mushroom tyrosinase and melanogenesis properties. The DT extract obtained using 50% ethanol at 50 °C exhibited the highest level of total phenol content and ABTS free-radical scavenging, and had the highest ferric-reducing power. The DT extract by water at RT exhibited the highest total flavonoid content. The DT extract by 50% ethanol at RT had the lowest IC_50_ for DPPH free-radical scavenging and mushroom tyrosinase inhibition ability. DT extracts showed no cytotoxicity to the B16F10 cells, and the RT + W and 50T + W exhibited better effects on anti-melanogenesis than the RT + 50E and 50T + 50E. The results in this study indicated that DT extracts may be a potential material in skincare.
